# MRI delta radiomics during chemoradiotherapy for prognostication in locally advanced cervical cancer

**DOI:** 10.1186/s12885-025-13509-1

**Published:** 2025-01-22

**Authors:** Kari S. Wagner-Larsen, Njål Lura, Ankush Gulati, Stian Ryste, Erlend Hodneland, Kristine E. Fasmer, Kathrine Woie, Bjørn I. Bertelsen, Øyvind Salvesen, Mari K. Halle, Noeska Smit, Camilla Krakstad, Ingfrid S. Haldorsen

**Affiliations:** 1 Mohn Medical Imaging and Visualization Centre (MMIV), Department of Radiology, Haukeland University Hospital, Jonas Lies vei 65, Bergen, 5021 Norway; 2 Section for Radiology, Department of Clinical Medicine, University of Bergen, Bergen, Norway; 3 Department of Obstetrics and Gynecology, Haukeland University Hospital, Bergen, Norway; 4 Department of Pathology, Haukeland University Hospital, Bergen, Norway; 5 Clinical Research Unit, Department of Clinical and Molecular Medicine, Norwegian University of Science and Technology, Trondheim, Norway; 6 Centre for Cancer Biomarkers (CCBIO), Department of Clinical Science, University of Bergen, Bergen, Norway; 7 Department of Informatics, University of Bergen, Bergen, Norway

**Keywords:** Uterine cervical neoplasms, Chemoradiotherapy, Magnetic resonance imaging, Delta-radiomics, Radiomics

## Abstract

**Background:**

Effective diagnostic tools for prompt identification of high-risk locally advanced cervical cancer (LACC) patients are needed to facilitate early, individualized treatment. The aim of this work was to assess temporal changes in tumor radiomics (delta radiomics) from T2-weighted imaging (T2WI) during concurrent chemoradiotherapy (CCRT) in LACC patients, and their association with progression-free survival (PFS). Furthermore, to develop, validate, and compare delta- and pretreatment radiomic signatures for prognostic modeling.

**Methods:**

A total of 110 LACC patients undergoing CCRT with MRI at baseline and mid-treatment were divided into training (cohort_T_: *n* = 73) and validation (cohort_V_: *n* = 37) cohorts. Radiomic features were extracted from tumors segmented on pre-CCRT and mid-CCRT T2WI and radiomic deltas (delta features) were computed. Two radiomic signatures for predicting PFS were constructed by least absolute shrinkage and selection operator (LASSO) Cox regression: Delta_rad_ (from delta features) and Pre-CCRT_rad_ (from pre-CCRT features). Prognostic performance of the radiomic signatures, 2018 International Federation of Gynecology and Obstetrics (FIGO) stage (I–IV), and baseline MRI-derived maximum tumor diameter (Tumor_max_: ≤2 cm; >2 and ≤ 4 cm; >4 cm) was evaluated by area under time-dependent receiver operating characteristics (tdROC) curves (AUC) in cohort_T_ and cohort_V_ (AUC_T_/AUC_V_). Mann–Whitney U tests assessed differences in radiomic delta features. PFS was evaluated using the Kaplan–Meier method with log-rank tests.

**Results:**

Delta_rad_ (AUC_T_/AUC_V_: 0.74/0.79) marginally outperformed Pre-CCRT_rad_ (0.72/0.75) for predicting 5-year PFS, and both signatures clearly surpassed that of FIGO (0.61/0.61) and Tumor_max_ (0.58/0.65). In total, four features within Delta_rad_ and Pre-CCRT_rad_ significantly differed in delta feature values between progressors and non-progressors, being consistently lower in progressors (*p* ≤ 0.03 for all). High Delta_rad_ and Pre-CCRT_rad_ radiomic scores were associated with poor PFS (*p* ≤ 0.04 for Delta_rad_ in cohort_T_/Pre-CCRT_rad_ in both cohorts; *p* = 0.11 for Delta_rad_ in cohort_V_).

**Conclusions:**

Delta- and pretreatment radiomic signatures equally allow early prognostication in LACC, outperforming FIGO stage and MRI-assessed maximum tumor diameter.

**Supplementary Information:**

The online version contains supplementary material available at 10.1186/s12885-025-13509-1.

## Background

Cervical cancer (CC) ranks as the fourth most common cancer and the fourth leading cause of cancer-related deaths among women globally [1]. Treatment strategies for CC are guided by disease stage at primary diagnosis, defined by the 2018 International Federation of Gynecology and Obstetrics (FIGO) staging system [2]. For large tumors and locally advanced CC (LACC) (FIGO IB3–IVA), the standard regimen typically involves cisplatin-based concurrent chemoradiotherapy (CCRT) followed by brachytherapy [2, 3]. The reported 5-year overall survival rate for patients with LACC is approximately 70% [4]. However, ~ one third of these patients experience recurrence, usually within two years after treatment [5]. Recurrent LACC poses significant treatment challenges, often with limited survival prospects [4]. Hence, promptly identifying patients at higher risk for disease recurrence or CCRT failure is essential to enable early decision support for more tailored treatment strategies and follow-up plans.

Pelvic magnetic resonance imaging (MRI) plays a pivotal role in the clinical management of LACC, assisting in staging, radiation treatment planning, and monitoring of therapeutic response [6]. In the last few years, MRI-based radiomic tumor profiling has been introduced, promoting the use of non-invasive imaging biomarkers for predicting risk factors, response to therapy, and survival in CC [7, 8]. This technique uses computational analysis to extract numerous quantitative imaging features, revealing mesoscopic tumor characteristics that are closely associated with tumor heterogeneity, clinical phenotype, and tumor aggressiveness [9]. However, most radiomic studies on CC typically focus on pretreatment imaging data only, inherently not capturing the dynamic tumor changes occurring during therapy. Delta radiomics quantifies temporal variations in tumor radiomic features [10]. This approach enables a more comprehensive characterization of treatment-induced tumor changes and disease evolution [11]. Recent studies have reported specific MRI radiomic features from tumors when undergoing CCRT, as biomarkers for early prediction of recurrence and therapeutic non-responsiveness in LACC [12–16]. Furthermore, MRI delta radiomic signatures, which incorporate multiple radiomic features into a model, have proven useful in predicting treatment responses across a variety of cancer types, including rectal cancer (undergoing chemoradiotherapy) [17, 18], breast cancer (chemotherapy) [19], and soft tissue sarcomas (radiotherapy +/- chemotherapy) [20]. The prognostic value of MRI delta radiomic signatures in CC is largely unknown, with only a few previous reports suggesting that such signatures may be linked to prognostic histopathological features and outcome [21, 22].

This study aimed to develop, validate, and compare a delta radiomic signature from T2-weighted imaging (T2WI) during CCRT against a pretreatment T2WI radiomic signature for early prediction of progression-free survival (PFS) in LACC patients. We also sought to explore how T2WI radiomic tumor features evolve during CCRT and examine the relationship between these changes and future disease progression.

## Methods

### Patients

**Fig. 1:**
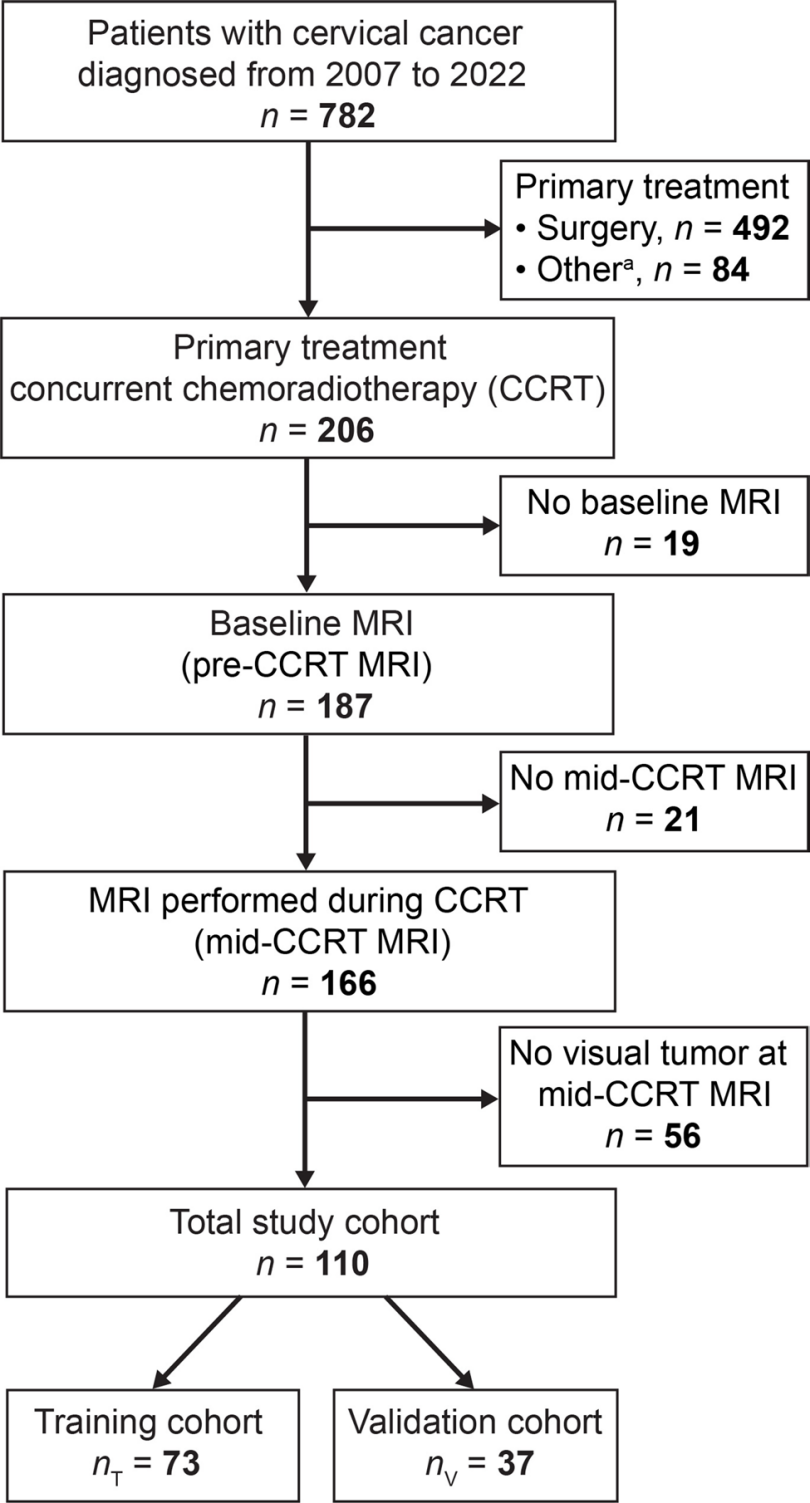
Study flowchart. The study is based on a cohort of patients with histologically verified cervical cancer (diagnosed during 2007–2022), who consented to participate (with a participation rate exceeding 95%). Patients included in the study cohort (*n* = 110) (2018 FIGO stage IB2–IVA) were all scheduled for primary treatment with CCRT followed by brachytherapy, had paired MRI examinations; at baseline (pre-CCRT MRI) and during CCRT (mid-CCRT MRI), and had visible tumors at both MRI scans. ^a^Chemotherapy or radiation therapy alone, neoadjuvant chemotherapy followed by surgery, or palliative treatment. CCRT, concurrent chemoradiotherapy; FIGO, International Federation of Gynecology and Obstetrics

This retrospective study on prospectively collected data was approved by the Regional Committee for Medical Research Ethics (2015/2333/REK vest). Written informed consent was obtained from all patients at the time of primary diagnosis. Patients admitted to Haukeland University Hospital during 2007–2022 with histologically confirmed CC were enrolled (*n* = 782). Inclusion criteria were (1) primary treatment plan comprising CCRT followed by brachytherapy, (2) paired MRI examinations: at baseline (pre-CCRT MRI) and during CCRT (mid-CCRT MRI), and (3) visible tumors at both MRI scans. A total of 110 LACC patients, diagnosed from June 2007 to October 2022, met the criteria. The patients were divided into training (cohort_T_: *n* = 73) and validation (cohort_V_: *n* = 37) cohorts in a 2:1 ratio (Fig. 1).

Clinical- and patient follow-up data were collected from medical records. Disease progression was defined as local recurrence/progression or new metastases during the follow-up period, confirmed by clinical examinations with biopsy or imaging (computed tomography (CT), MRI, and/or ^18^F-fluorodeoxyglucose positron emission tomography with CT (FDG-PET/CT). Patients with imaging indicative of progression (such as tumor growth or new lesions/new FDG-PET/CT positive lesions without history of other malignancies) were categorized as “progression”, even without histological confirmation. Ambiguous imaging findings without a positive biopsy were categorized as “no progression”. PFS was defined as time from primary diagnosis until disease progression. Date of last follow-up was June 2023. The median (mean) [IQR] time to progression was 14 (20) [7–25] months. Non-progressors had a median (mean) [IQR] follow-up of 65 (76) [27–112] months.

The study cohort (*n* = 110) had similar clinical- and pathological patient characteristics as the entire CC cohort treated with CCRT during the same time period at our hospital (*n* = 206) (Suppl. Table 1).

### Primary treatment and imaging schedule

All patients underwent CCRT with curative intent and were scheduled for subsequent intracavitary or interstitial brachytherapy. The pre-CCRT MRI, conducted prior to treatment onset, facilitated staging and external beam radiation therapy (EBRT) planning. After ~ 4 weeks of CCRT, the mid-CCRT MRI was performed before initiation of brachytherapy, enabling tailored brachytherapy dosage adjustments according to the residual tumor size. By mid-CCRT MRI, 80% (88/110) of the patients had received four or five weekly doses of Cisplatin (40mg/m^2^), and 93% (102/110) had undergone ~ 20 sessions of pelvic EBRT at 1.8 Gy per fraction (Table 1). The median (mean) [IQR] duration from start of treatment to mid-CCRT MRI was 29 (29) [27–31] days. Details of the primary treatment administered up to the mid-CCRT MRI as well as the cumulative primary treatment are given in Table 1. Brachytherapy was administered to 99% (109/110) of the patients; one patient was considered unsuitable for the procedure due to technical challenges (i.e., obscured external os of the cervical canal caused by extensive tumor growth) (Table 1).

**Table 1:** Primary treatment administered up to mid-CCRT MRI and cumulative primary treatment given

Treatment type and regimen	By mid-CCRT MRI, *n* (%)	Cumulative treatment, *n* (%)
**Chemotherapy**		
Cisplatin (doses)		
1	1 (1)	1 (1)
2	2 (2)	2 (2)
3	10 (9)	1 (1)
4	48 (44)	12 (11)
5	40 (36)	69 (63)
6	5 (5)	21 (19)
3 doses + Pembroluzimab/Placebo	1 (1)	1 (1)
2 doses + Carboplatin (2 doses)	1 (1)	
2 doses + Carboplatin (3 doses)		1 (1)
Carboplatin (4 doses)	2 (2)	2 (2)
**External beam radiation therapy fraction**		
1.8 Gy (~ 15 sessions)	1 (1)	
1.8 Gy (~ 20 sessions)	102 (93)	
1.8 Gy (~ 25 sessions)	7 (6)	
1.8 Gy (25 sessions)		110 (100)
**Primary radiation boost** ^a^		
Yes		87 (79)
No		23 (21)
**Hyperthermia** ^a^		
Yes		9 (8)
No		101 (92)
**Brachytherapy**		
Intracavitary		91 (83)
Interstitial		18 (16)
None		1 (1)

CCRT, concurrent chemoradiotherapy; Gy, gray

^a^Administered in part during mid-CCRT MRI

### MRI acquisition

Pre-CCRT MRI was performed on scanners from three different vendors (Siemens Healthineers, Germany; GE Healthcare, USA; Philips Healthcare, Netherlands), using 1.5T (60/110 patients) and 3.0T (50/110 patients) systems across four hospitals in Western Norway. Mid-CCRT MRI was performed in one hospital using two vendors’ (Siemens Healthineers, GE Healthcare) 1.5T (79/110 patients) and 3.0T (31/110 patients) systems. The MRI examinations were conducted as part of standard clinical assessments. Imaging protocols varied among scanners and institutions; however, all protocols adhered to the European Society of Urogenital Radiology (ESUR) guidelines [6]. Axial/axial oblique (perpendicular to the long axis of the cervix) T2WI was chosen for radiomic analysis as T2WI was consistently acquired as a part of the standard protocols across all institutions at both time points.

The pre-CCRT MRIs comprised axial/axial oblique, sagittal, and coronal/coronal oblique (perpendicular to the short axis of the cervix) T2WI. In addition, 109/110 included axial T1-weighted imaging (T1WI), 84/110 diffusion-weighted imaging (DWI), and 28/110 contrast-enhanced T1WI (CE T1WI). For the mid-CCRT MRIs, 30/110 followed a protocol tailored for the presence of a brachytherapy applicator, featuring axial oblique T2WI and 3D axial oblique T2WI with reformatted sagittal and coronal views. The other 80/110 featured axial/axial oblique and sagittal T2WI. Of these, 79/80 also had coronal/coronal oblique T2WI and axial T1WI, while 60/80 included DWI, and only 6/80 CE T1WI. Of the mid-CCRT MRIs, 58/110 were performed with a cervical sleeve, and 28/110 of the examinations also included a brachytherapy applicator.

The MRI scans in both cohort_T_ and cohort_V_ were similarly distributed across institutions, vendors, and field strength at both pre-CCRT and mid-CCRT time points. A detailed overview of MRI acquisition parameters is given in Suppl. Table 2.

### Tumor segmentation and image analyses

The MRI examinations were de-identified prior to independent review by radiologists, who were blinded to clinical information. The whole volume of the tumors was manually segmented on both pre- and mid-CCRT MRIs using axial oblique (when available) or axial T2WI, excluding any cervical/vaginal devices when present. DWI series and CE T1WI aided in verifying tumor borders when available. One radiologist (K.W.L) with 13 years of pelvic MRI experience segmented the tumors at both time points in all the cases. To assess interobserver reproducibility for tumor segmentations, a second radiologist (N.L) with 8 years of pelvic MRI experience, manually segmented tumors at both time points in 28 randomly chosen cases. The segmentations were performed using the open-source software ITK-SNAP (version 3.6.0) [23].

At baseline, the maximum tumor diameter, regardless of plane, was measured on T2WI by three radiologists. Overall, five radiologists (K.W.L, N.L, A.G, S.R, I.J.M), each having 3 to 20 years of experience in reading pelvic MRI, contributed to the study. A consensus measurement was derived using the median of the tumor size values. At mid-CCRT, the maximum tumor diameter, irrespective of plane, was measured on T2WI by one radiologist (K.W.L).

### Radiomic feature extraction

Image data and tumor masks were loaded using the open-source, Python-based package Imagedata [24]. Before feature extraction, the axial (oblique) T2WI images were normalized to a standardized voxel intensity distribution; each data set was divided by its own average and multiplied by 100:

$$\:f\leftarrow\:f\bullet\:\frac{100}{\stackrel{-}{f}}$$

Radiomic features were extracted using PyRadiomics (version 3.1.0) for Python (version 3.6.13) [25]. Feature extraction was performed in 3D. Default Pyradiomics settings were applied, with the following exceptions: a fixed bin width of 10 was used for discretization of the intensity values and the interpolator was specified as “sitkLinear”. First-order features describing the signal intensity, e.g., mean, median, range, and percentiles, were excluded due to the arbitrary nature of the T2WI signal. Shape features, including tumor volume, were omitted to reduce the impact of tumor size, a known prognostic factor in LACC [26]. This allowed for a focus on a broader array of less size-dependent radiomic features, providing deeper insights into intrinsic tumor characteristics.

In total, 82 radiomic features were extracted at both pre-CCRT and mid-CCRT time points, including 7 first-order features and 75 textural features comprising five classes: Gray Level Co-occurrence Matrix (GLCM: *n* = 24), Gray Level Dependence Matrix (GLDM: *n* = 14), Gray Level Run Length Matrix (GLRLM: *n* = 16), Gray Level Size Zone Matrix (GLSZM: *n* = 16), and Neighboring Gray Tone Difference Matrix (NGTDM: *n* = 5) (Suppl. Table 3).

To reduce the variability arising from different MRI scanners and settings, we implemented a two-step normalization process of the radiomic features. Concatenating the radiomics data from both pre-CCRT and mid-CCRT MRIs, a linear regression model using repetition time (TR), time to echo (TE), flip angle (FA), slice thickness, number of excitations (NEX), anisotropy, voxel volume, field of view (FOV), field strength, and manufacturer as predictive variables (Suppl. Table 2) was fitted to each of the radiomic features. To account for the influence of various scanner protocol parameters, the linear prediction from each of the radiomic features was subtracted, yielding a set of remaining residuals that were used for statistical modeling. Finally, the radiomic features were z-normalized to have a mean of zero with a standard deviation of one.

### Selecting robust features

The Dice similarity coefficient [27] was employed to assess the agreement of the tumor segmentations delineated by the two radiologists. This coefficient ranges from 0, signifying no overlap, to 1, which represents perfect congruence. The two radiologists exhibited good agreement on the pre-CCRT segmentations with a Dice score of 0.85, while the agreement on the mid-CCRT segmentations was moderate, reflected by a Dice score of 0.57.

Based on the segmentations performed by both radiologists, we calculated the intraclass correlation coefficient (ICC) for every radiomic feature. Due to the middling Dice score from the mid-CCRT segmentations, we opted for strict robustness standards. Only features with an ICC > 0.75 and a lower bound confidence interval (CI) of ≥ 0.60 in both pre-CCRT and mid-CCRT MRIs were considered robust and selected for further statistical analyses. Consequently, the final radiomic dataset comprised 26 features (4 GLCM, 8 GLDM, 8 GLRLM, and 6 GLSZM) (Suppl. Table 3).

### Delta radiomic feature computation

The change in each radiomic feature between the pre-CCRT and mid-CCRT time points, termed (delta) Δfeature, was computed as follows:

Δfeature = feature value at mid-CCRT MRI − feature value at pre-CCRT MRI.

### Model development

**Fig. 2:**
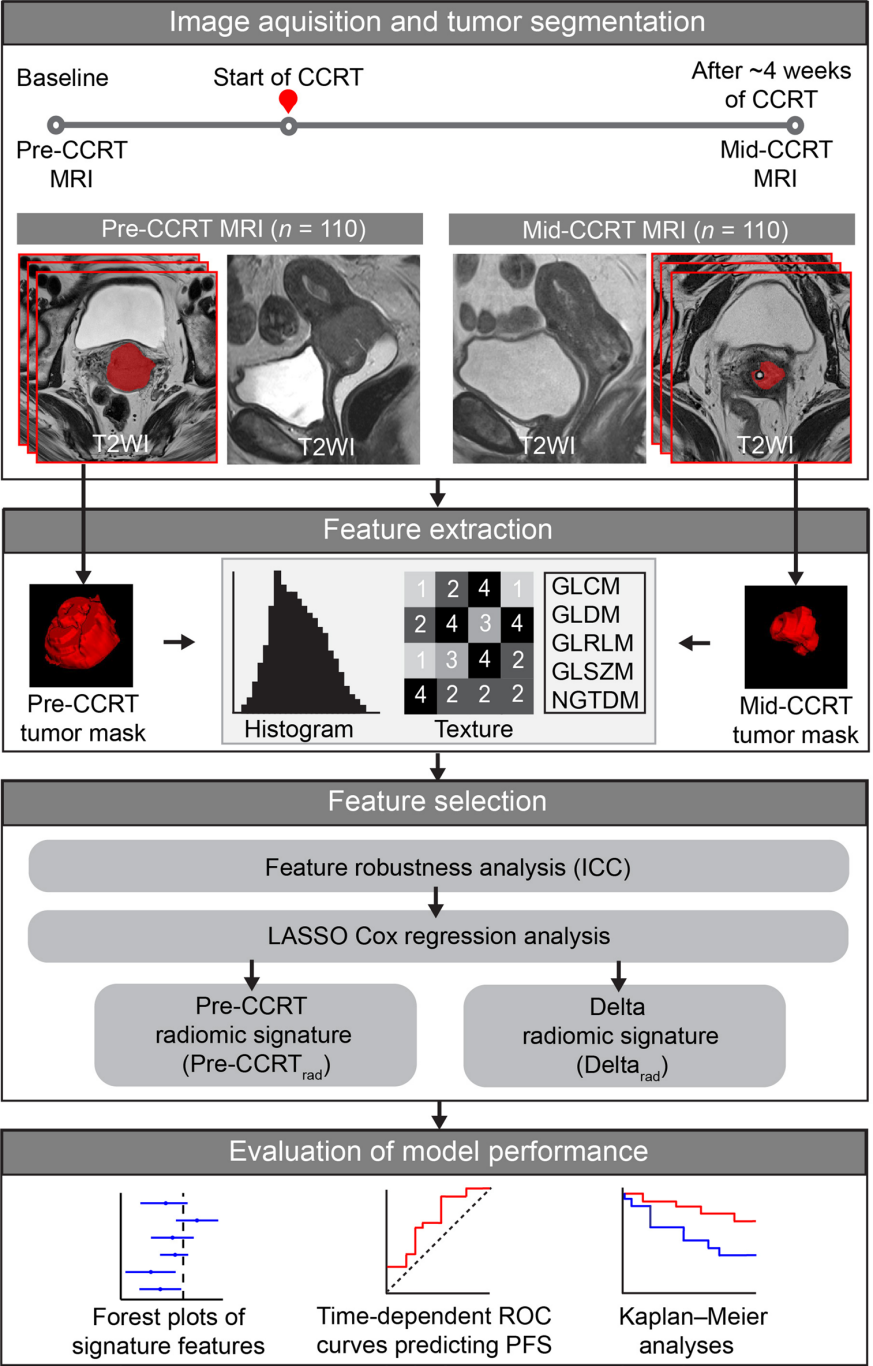
Radiomics analysis workflow. Radiomic features were extracted from whole-volume tumor masks at pre- and mid-CCRT T2WI MRIs. Only features with ICC > 0.75 and a lower CI ≥ 0.60 in both scans (based on segmentations performed by two radiologists in 28 overlapping cases) were retained. LASSO Cox statistics were used to generate two radiomic signatures for predicting PFS: Delta_rad_, based on Δfeatures (i.e., the change between mid- and pre-CCRT features) and Pre-CCRT_rad_, using pre-CCRT radiomic features. CCRT, concurrent chemoradiotherapy; CI, confidence interval; GLCM, Gray Level Co-occurrence Matrix; GLDM, Gray Level Dependence Matrix; GLRLM, Gray Level Run Length matrix; GLSZM, Gray Level Size Zone Matrix; ICC, intraclass correlation coefficient; LASSO, least absolute shrinkage and selection operator; NGTDM, Neighboring Gray Tone Difference Matrix; PFS, progression-free survival; ROC, receiver operating characteristics; T2WI, T2-weighted imaging

Least absolute shrinkage and selection operator (LASSO) Cox regression [28] for prediction of PFS was applied to cohort_T_ for feature selection and the construction of radiomic signatures. The regularization parameter lambda (λ) was optimized by leave-one-out cross-validation. Two radiomic signatures were developed: one from Δfeatures (Delta_rad_) and another from pre-CCRT features (Pre-CCRT_rad_). Radiomic scores were subsequently derived by linearly combining the final selected features, each multiplied by its corresponding coefficient. The signatures, derived in cohort_T_, were validated in cohort_V_. The complete radiomics analysis workflow is illustrated in Fig. 2.

### Statistical analyses

Statistical analyses were conducted using R 4.2.3 (R Core Team, 2023) [29] and STATA 17.0 (StataCorp. 2021) [30]. The dataset was divided into cohort_T_ and cohort_V_ through supervised stratification using scikit-learn’s train_test_split (version 0.24.2) [31]. Stratification was based on disease progression during follow-up, follow-up time, and MRI field strength. ICCs were calculated using the “ICC” R-package. Differences in clinicopathological patient features for the cohorts were assessed using Mann–Whitney U tests for continuous variables and Fischer’s exact tests for categorical variables. LASSO Cox used the “glmnet” R-package. Correlations between MRI-derived tumor diameter and the features within Delta_rad_ and Pre-CCRT_rad_ were assessed with Spearman’s rank tests. Wilcoxon signed-rank tests were used to evaluate the differences in MRI-derived tumor diameter from pre- to mid-CCRT, as well as temporal changes in radiomic signature features. Median Δfeature differences (location shifts) between patients who did/did not develop progression, at any time during follow-up, were evaluated by Mann–Whitney U tests. The “forestplot” R-package was used to visualize these differences. The “timeROC” R-package was used to compute and compare the area under the time-dependent receiver operating characteristics (tdROC) curves (AUC) for predicting 5-year PFS in cohort_T_ (AUC_T_) and cohort_V_ (AUC_V_). Optimal cutoffs for Delta_rad_ and Pre-CCRT_rad_ in cohort_T_ were identified from tdROC curves using the “survivalROC” R-package’s Youden Index. Patients were then grouped based on high or low radiomic scores. Differences in PFS between the groups were analyzed with the Kaplan–Meier method and log-rank tests. Reported *p* values were considered statistically significant when < 0.05.

## Results

### Patient demographics

Median patient age at primary diagnosis was 48 years [IQR: 39–57]. FIGO stages included: IB2/IB3 (*n* = 8), IIA (*n* = 6), IIB (*n* = 32), IIIB (*n* = 7), IIIC (*n* = 50), and IVA (*n* = 7). At baseline, the median MRI-derived maximum tumor diameter was 5.2 cm [IQR: 4.4–6.7]. Maximum tumor diameter was significantly lower, measuring a median of 2.9 cm [IQR: 2.3–4.3], at mid-CCRT MRI (*p* < 0.001). Patients developing progression had larger tumors (median) than non-progressors both at baseline (6.5 cm vs. 4.8 cm) and at mid-CCRT MRI (3.5 cm vs. 2.6 cm) (*p* ≤ 0.002 for both), but similar relative size reduction (median of 36% vs. 41%, *p* = 0.63). The size reduction was ≥ 30% [IQR: 38–54%] in 70% (77/110) of the patients, while the remaining 30% (33/110) experienced < 30% [IQR:10–24%] tumor shrinkage, with equal distribution among progressors and non-progressors (*p =* 0.83).

During follow-up, 35 patients experienced disease progression (8 local/pelvic, 16 abdominal, and 11 distant); 29 of these eventually died from CC (Table 2).

**Table 2:** Clinicopathological characteristics of the patients with separate figures for the training and validation cohorts

	Total study cohort (*n* = 110)	Training cohort (*n* = 73)	Validation cohort (*n* = 37)	*p*
Age, median (IQR)	48 (39–57)	46 (37–55)	51 (45–59)	0.06
BMI, kg/m^2^, median (IQR)	25 (22–28)	25 (22–28)	25 (22–28)	0.73
Menopausal status, *n* (%)				**0.01**
Pre- /perimenopausal	67 (61)	51 (70)	16 (43)	
Postmenopausal	43 (110)	22 (30)	21 (57)	
Maximum tumor diameter at pre-CCRT MRI^a^, *n* (%)				1.00
≤ 2 cm	2 (2)	1 (1)	1 (3)	
> 2 and ≤ 4 cm	23 (21)	15 (21)	8 (22)	
> 4 cm	85 (77)	57 (78)	28 (76)	
Maximum tumor diameter at mid-CCRT MRI^a^, *n* (%)				0.85
≤2 cm	16 (15)	11 (15)	5 (14)	
>2 and ≤ 4 cm	62 (56)	42 (58)	20 (54)	
>4 cm	32 (29)	20 (27)	12 (32)	
FIGO stage, *n* (%)				0.51
I	8 (7)	5 (7)	3 (8)	
II	38 (35)	25 (34)	13 (35)	
III	57 (52)	40 (55)	17 (46)	
IV	7 (6)	3 (4)	4 (11)	
Histologic type, *n* (%)				0.13
Squamous cell carcinoma	91 (83)	64 (88)	27 (73)	
Adenocarcinoma	17 (16)	8 (11)	9 (24)	
Other^b^	2 (2)	1 (1)	1 (3)	
Histologic grade, *n* (%)				0.76
1&2	51 (46)	35 (48)	16 (43)	
3	16 (15)	12 (16)	4 (11)	
Not recorded	43 (39)	26 (36)	17 (46)	
Progression, *n* (%)				0.83
Yes	35 (32)	24 (33)	11 (30)	
No	75 (68)	49 (67)	26 (70)	
Dead from cervical cancer, *n* (%)				0.82
Yes	29 (26)	20 (27)	9 (24)	
No	81 (74)	53 (73)	28 (76)	

*P* values refer to test of differences between the training- and validation cohort (Mann–Whitney U test for continuous variables and Fisher’s exact test for categorical variables). Significant *p* values are given in **bold**

^a^MRI-derived maximum tumor diameter, measured regardless of plane, later grouped into three categories

^b^Undifferentiated carcinoma or inconclusive biopsy.

BMI, body mass index; CCRT, concurrent chemoradiotherapy; FIGO, International Federation of Gynecology and Obstetrics; IQR, interquartile range

Cohort_T_ and cohort_V_ had similar clinicopathological patient characteristics, except for a slightly higher percentage of pre-/perimenopausal women in cohort_T_ (in 70% (51/73) vs. 43% (16/37) in cohort_V_, *p* = 0.01) (Table 2).

### Feature selection and radiomic signature construction

Detailed formulas for the radiomic signatures are provided in Table 3. Specifically, from the delta radiomics dataset, six Δfeatures (f1–f2, f4–f5, f7, f10) were selected using the LASSO Cox method to form the Delta_rad_ signature for PFS prediction. These encompassed GLCM (*n* = 2), GLDM (*n* = 2), and GLSZM (*n* = 2) features (Table 3). In a similar manner, the Pre-CCRT_rad_ signature was developed using the pre-CCRT dataset. This signature comprised seven features (f3–f9): GLDM (*n* = 3), GLRLM (*n* = 1), and GLSZM (*n* = 3). The two signatures shared three common radiomic features (f4, f5, f7) (Table 3).

**Table 3:** Radiomic features selected by LASSO Cox for PFS prediction using features (Deltarad) and pre-CCRT features (Pre-CCRTrad)

Δ			
Radiomic features	(Abbreviation)	Delta_rad_	Pre-CCRT_rad_
GLCM Autocorrelation	(f1)	-0.020	
GLCM Inverse Variance	(f2)	0.006	
GLDM Dependence Non-Uniformity Normalized	(f3)		-0.210
GLDM Dependence Variance	(f4)	-0.379	0.583
GLDM Gray Level Variance	(f5)	0.044	-0.094
GLRLM Long Run Emphasis	(f6)		-0.270
GLSZM Size Zone Non-Uniformity Normalized	(f7)	-0.239	0.132
GLSZM Small Area Emphasis	(f8)		0.142
GLSZM Small Area High Gray Level Emphasis	(f9)		0.128
GLSZM Zone%	(f10)	-0.350	
Regularization parameter λ		0.033	0.019
Radiomic signature calculation formulas			
Delta_rad_ = (-0.020xΔf1) + (0.006xΔf2) + (-0.379xΔf4) + (0.044xΔf5) + (-0.239xΔf7) + (-0.350xΔf10)			
Pre-CCRT_rad_ = (-0.210xf3) + (0.583xf4) + (-0.094xf5) + (-0.270xf6) + (0.132xf7) + (0.142xf8) + (0.128xf9)			

CCRT, concurrent chemoradiotherapy; GLCM, Gray Level Co-occurrence Matrix; GLDM, Gray Level Dependence Matrix; GLRLM, Gray Level Run Length Matrix; GLSZM, Gray Level Size Zone Matrix; LASSO, Least absolute shrinkage and selection operator

**Table 4:** Temporal radiomic changes from pre-CCRT to mid-CCRT MRI for the features comprising Delta_rad_ and Pre-CCRT_rad_

	Pre-CCRT		Mid-CCRT			
Radiomic features	Median	(IQR)	Median	(IQR)	Change	*p*
GLCM Autocorrelation^a^	-0.123	(1.032)	-0.259	(0.781)	↓	0.11
GLCM Inverse Variance^a^	0.163	(1.123)	0.084	(1.196)	↓	0.06
GLDM Dependence Non-Uniformity Normalized^b^	-0.388	(0.976)	0.087	(1.174)	**↑**	**< 0.001**
GLDM Dependence Variance^a, b^	0.277	(1.349)	-0.396	(0.945)	**↓**	**< 0.001**
GLDM Gray Level Variance^a, b^	-0.248	(0.688)	-0.093	(0.438)	↑	0.08
GLRLM Long Run Emphasis^b^	0.104	(0.793)	-0.177	(0.493)	**↓**	**< 0.001**
GLSZM Size Zone Non-Uniformity Normalized^a, b^	0.112	(1.309)	-0.261	(1.143)	**↓**	**0.02**
GLSZM Small Area Emphasis^b^	0.155	(1.200)	-0.125	(1.055)	**↓**	**0.046**
GLSZM Small Area High Gray Level Emphasis^b^	-0.024	(1.393)	-0.365	(0.783)	**↓**	**0.003**
GLSZM Zone Percentage^a^	-0.183	(0.768)	-0.097	(0.920)	**↑**	**0.03**

*P* values refer to the Wilcoxon signed rank test. Significant *p* values are given in **bold**

^a^Features within Delta_rad_

^b^Features within Pre-CCRT_rad_

CCRT, concurrent chemoradiotherapy; GLCM, Gray Level Co-occurrence Matrix; GLDM, Gray Level Dependence Matrix; GLSZM, Gray Level Size Zone Matrix; IQR, Interquartile range (given as Q3-Q1)

Pre-CCRT values of seven (f1–f4 and f8–f10) of the in total ten features within both Delta_rad_ and Pre-CCRT_rad_ demonstrated weak to moderate correlations with baseline MRI-derived maximum tumor diameter (*r*_S_ = − 0.48–0.41, *p* ≤ 0.03 for all). Conversely, three features (f5–f7) were not significantly correlated (*r*_S_=0.03–0.18, *p* ≥ 0.06 for all) (Suppl. Table 4). In Delta_rad_, mid-CCRT values for four (f2, f4, f5, and f10) out of the six features were weakly to moderately correlated with tumor diameter at mid-CCRT MRI (*r*_S_ = − 0.54–0.51, *p* ≤ 0.007 for all), while the remaining two features (f1 and f7) were not (*r*_S_=0.0002 and − 0.002, *p* ≥ 0.99 for both) (Suppl. Table 4).

### Performance of the radiomic signatures for predicting 5-year PFS

**Fig. 3:**
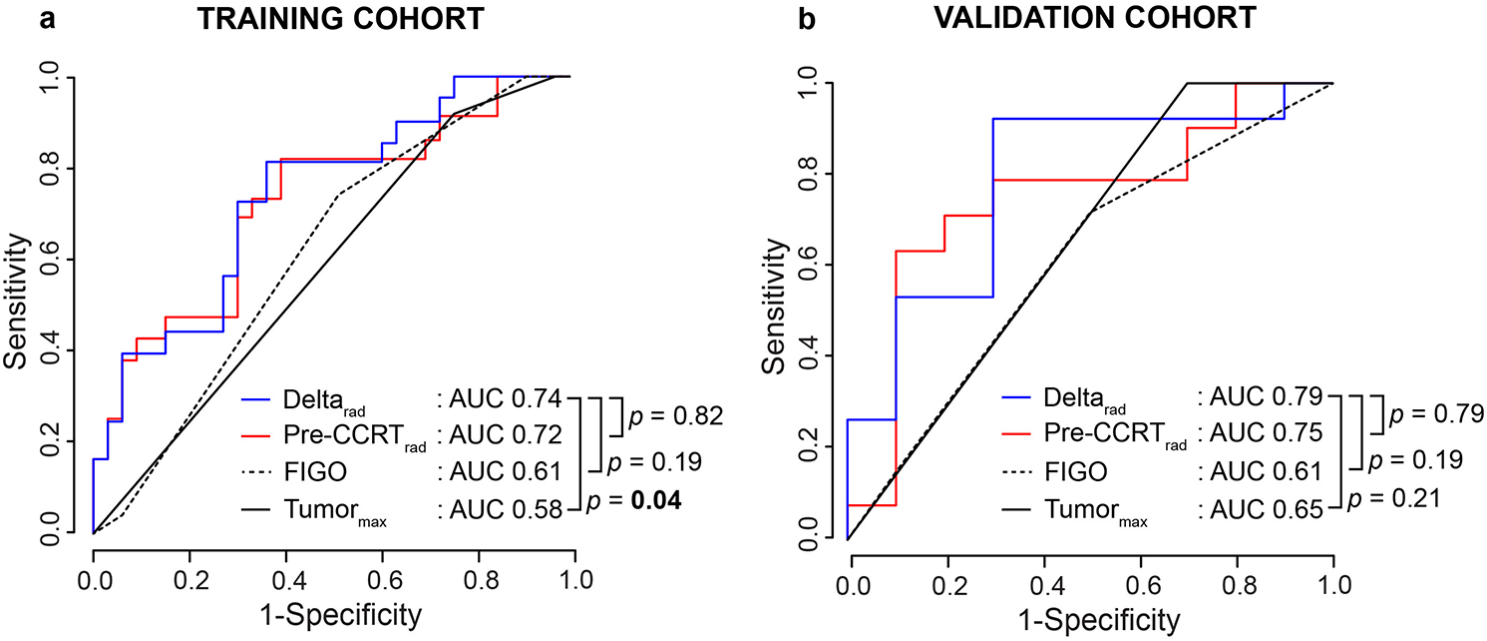
Time-dependent receiver operating characteristics (tdROC) curves for predicting 5-year progression-free survival (PFS) based on the radiomic signatures Delta_rad_ and Pre-CCRT_rad_, FIGO stage (I–IV), and MRI-derived maximum tumor diameter at pre-CCRT (Tumor_max_: ≤2; >2 and ≤ 4; >4 cm) in the training (**a**) and validation (**b**) cohorts. *P* values refer to the test of equal area under the tdROC curves (AUC). Significant *p* values are given in **bold**. CCRT, concurrent chemoradiotherapy; FIGO, International Federation of Gynecology and Obstetrics

For predicting 5-year PFS, Delta_rad_ yielded AUC_T_/AUC_V_ of 0.74/0.79, marginally surpassing Pre-CCRT_rad_ yielding AUC_T_/AUC_V_ of 0.72/0.75 (with no significant difference between AUC_T_/AUC_V_: *p* ≥ 0.79 in both cohorts) (Fig. 3). Both signatures demonstrated higher performance metrics for 5-year PFS prediction than FIGO stage (I–IV), with AUC_T_/AUC_V_ of 0.61/0.61, and baseline MRI-derived maximum tumor diameter (Tumor_max_: ≤2 cm; >2 and ≤ 4 cm; >4 cm), with AUC_T_/AUC_V_ of 0.58/0.65 (Fig. 3). Notably, Delta_rad_ significantly outperformed Tumor_max_ in cohort_T_ (*p* = 0.04) with a similar tendency in cohort_V_ (*p* = 0.21). Delta_rad_ also tended to outperform FIGO in predicting 5-year PFS across both cohorts (*p* = 0.19 for both) (Fig. 3). Tumor_max_ yielded comparable performance metrics for 5-year PFS prediction to those of the manually segmented tumor volume at baseline, however outperformed both relative change in MRI-measured maximum tumor diameter and in tumor volume (Suppl. Table 5).

**Fig. 4:**
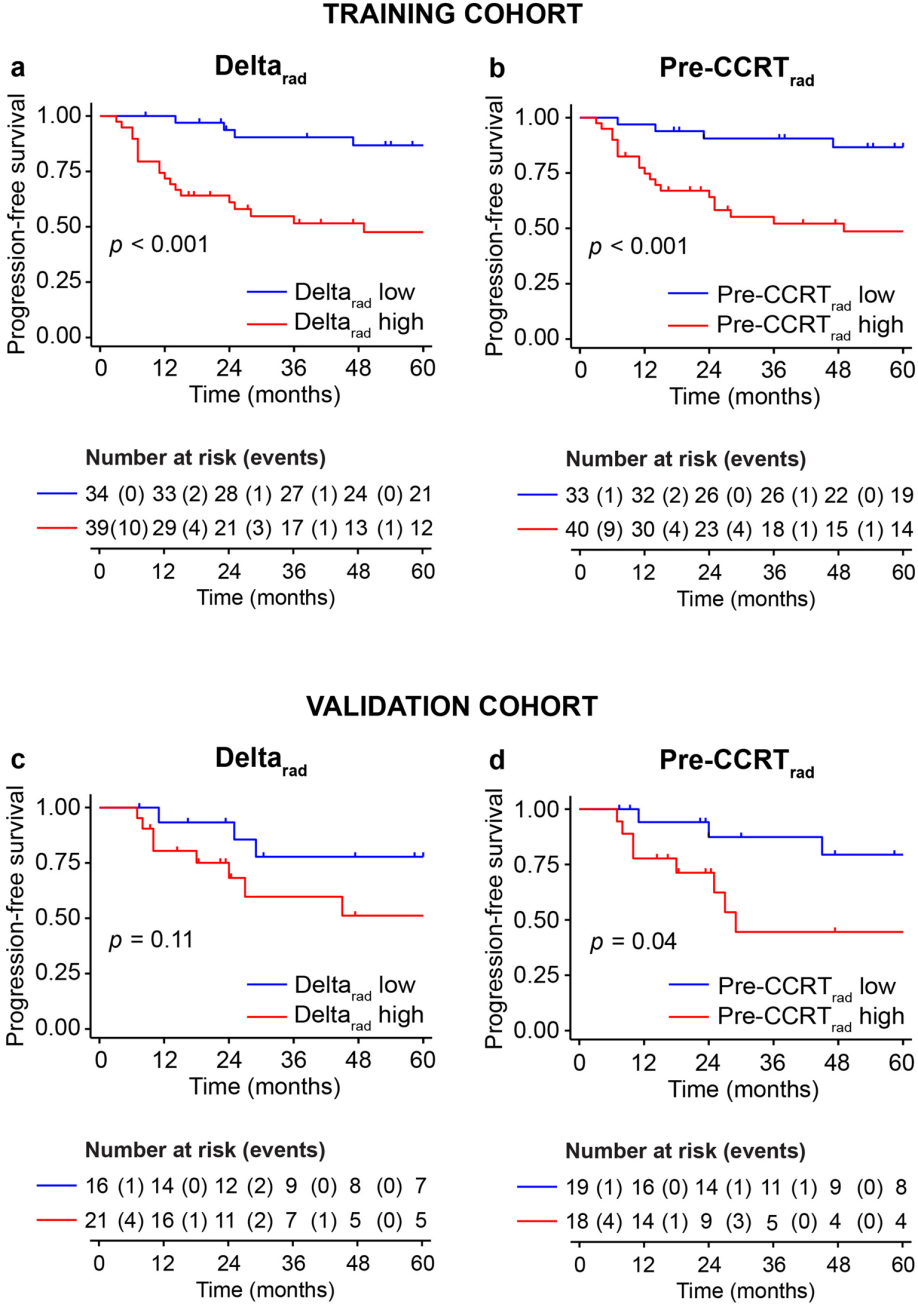
Kaplan–Meier curves depicting progression-free survival (PFS) rates among locally advanced cervical cancer (LACC) patients with low- and high radiomic scores for the radiomic signatures Delta_rad_ and Pre-CCRT_rad_ in the training (**a** and **b**) and validation (**c** and **d**) cohorts. The optimal cut-off values for the radiomic signatures were determined by time-dependent receiver operating characteristics (tdROC) curve analysis in the training cohort using Youden Index

A high Delta_rad_ radiomic score was associated with lower PFS in cohort_T_ (*p* < 0.001) and showed a similar trend in cohort_V_ (*p* = 0.11) (Fig. 4a, c). Patients with a high Pre-CCRT_rad_ score demonstrated significantly poorer PFS in both cohort_T_ and cohort_V_ (*p* < 0.001 and *p* = 0.04, respectively) (Fig. 4b, d).

### Delta radiomics

**Fig. 5:**
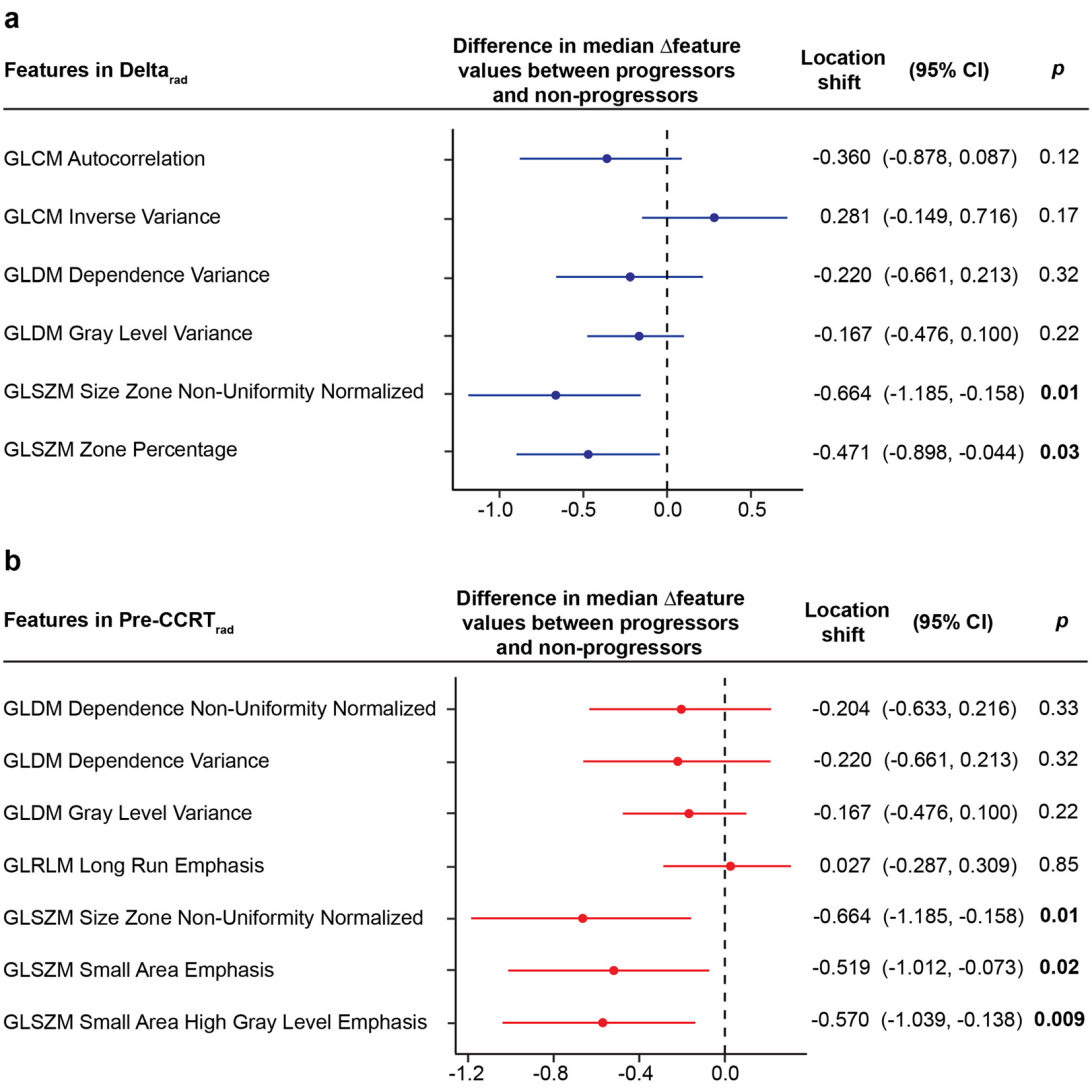
Forest plots of the estimated difference in median Δfeature (location shift) between progressors and non-progressors of the radiomic features included in the (**a**) Delta_rad_ and (**b**) Pre-CCRT_rad_ signatures. *P* values refer to the Mann-Whitney U test. Significant *p* values are given in **bold**. CCRT, concurrent chemoradiotherapy; CI, confidence interval; GLCM, Gray Level Co-occurrence Matrix; GLDM, Gray Level Dependence Matrix; GLRLM, Gray Level Run Length matrix; GLSZM, Gray Level Size Zone Matrix

**Fig. 6:**
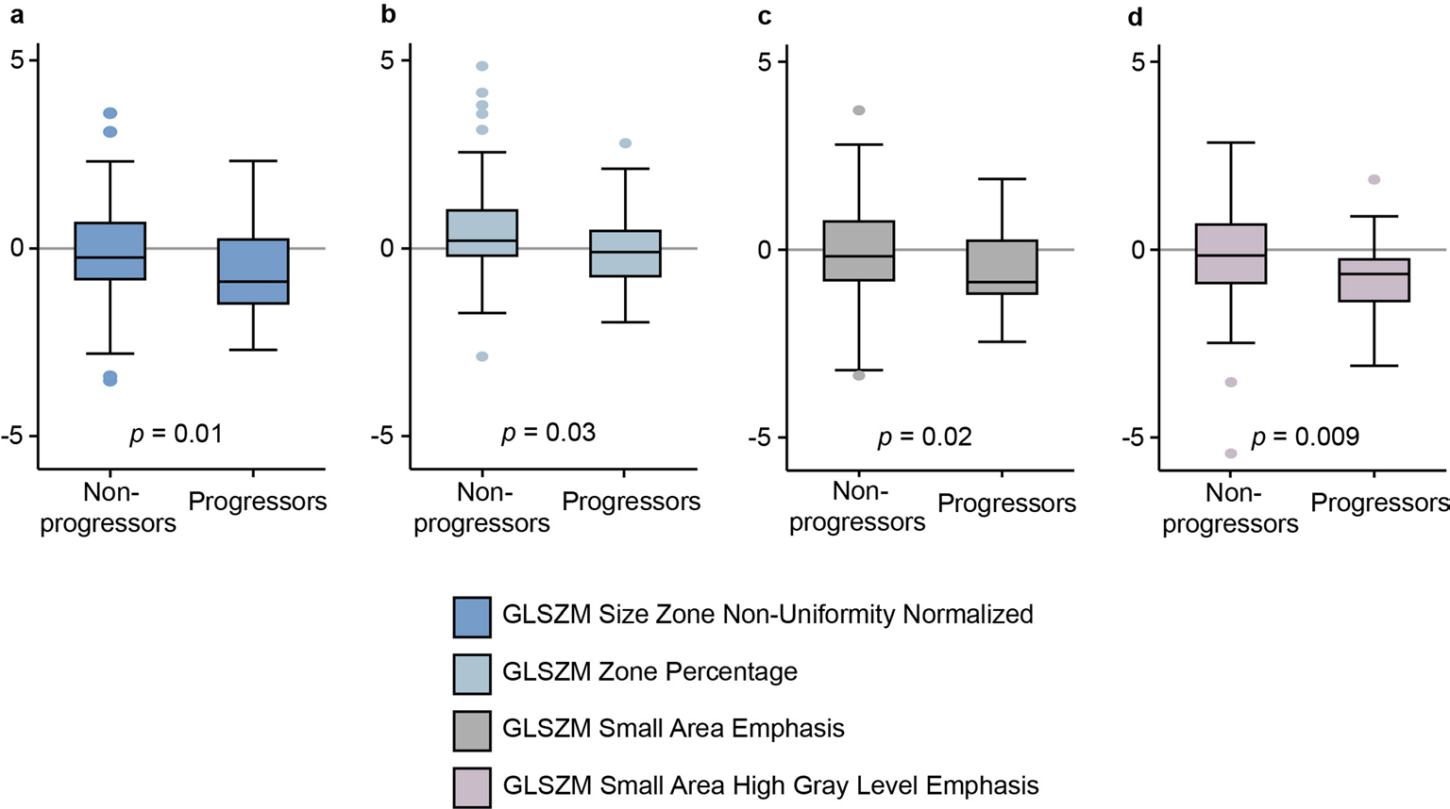
Box plot depicting significant differences between progressors and non-progressors for the median (IQR) [range] Δfeature values: (**a**) GLSZM Size Zone Non-Uniformity Normalized, (**b**) GLSZM Zone%, (**c**) GLSZM Small Area Emphasis, and (**d**) GLSZM Small Area High Gray Level Emphasis. CI, confidence interval; GLSZM, Gray Level Size Zone Matrix; IQR, interquartile range

Out of the ten features encompassing Delta_rad_ and Pre-CCRT_rad_, seven exhibited significant changes from pre-CCRT to mid-CCRT MRI (*p* ≤ 0.046 for all), with the rest showing a strong similar trend (*p* ≤ 0.11 for all) (Table 4). Most features (7/10) decreased during this time period, while three increased (Table 4). Four features within Delta_rad_ and Pre-CCRT_rad_ —GLSZM Size Zone Non-Uniformity Normalized (in both), GLSZM Zone% (Delta_rad_), and GLSZM Small Area Emphasis and GLSZM Small Area High Gray Level Emphasis (Pre-CCRT_rad_) —showed not only significant temporal changes but also distinctly different Δfeature values between progressors and non-progressors (Fig. 5), consistently presenting lower negative values in progressors (*p* ≤ 0.03 for all) (Fig. 6 and Suppl. Table 6).

## Discussion

This pioneering study is the first to develop and validate a delta radiomic MRI signature for prognostication in LACC patients undergoing CCRT, and to evaluate its prognostic potential compared with a pretreatment radiomic signature. We found that both the delta- and pretreatment radiomic signatures, derived from T2WI, equally enhance early prognostic assessment in LACC. Remarkably, these radiomic signatures outperformed conventional prognostic markers such as 2018 FIGO stage and baseline MRI-derived maximum tumor size for predicting PFS. The incorporation of delta- and pretreatment radiomic signatures into clinical practice has the potential to support more informed individualized treatment decisions and follow-up strategies in LACC, ultimately contributing to better patient outcomes.

Advanced imaging biomarkers are central for non-invasively identifying LACC patients at increased risk of disease recurrence or non-responsiveness to treatment. While tumor radiomic features assessed before treatment offer valuable insights into potential therapeutic outcomes, their inherent limitation lies in not being able to incorporate dynamic radiomic changes caused by CCRT. Delta radiomics addresses this limitation by analyzing temporal change in radiomic features [11]. Our study underscores the substantial prognostic potential of delta- and pretreatment radiomic signatures for early assessment of treatment response in LACC patients. With AUCs for 5-year PFS prediction of 0.74/0.79 for the delta radiomic signature (Delta_rad_) and 0.72/0.75 for the pretreatment signature (Pre-CCRT_rad_) in the training/validation cohorts, they clearly surpass traditional prognostic markers such as FIGO stage (I–IV) and baseline MRI-derived maximum tumor diameter (≤ 2; >2 and ≤ 4; >4 cm), which yielded AUCs of 0.61/0.61 and 0.58/0.65, respectively. Our AUCs of 0.72–0.79 are in line with prior studies on pretreatment T2WI-based radiomic signatures in LACC patients undergoing CCRT. These studies reported AUCs of 0.71/0.70 (training/validation cohorts) for predicting treatment response (≥ 30% tumor size reduction post-4 weeks CCRT [32], and an AUC of 0.88 (training cohort) for 3-year overall survival prediction [33].

One small study has explored delta radiomic signatures in LACC patients (*n* = 39) receiving CCRT [22]. Their model, using 2–3 delta T2WI radiomic features (from initial- and final brachytherapy MRIs), yielded AUCs of 0.70–0.71 (training cohort) for predicting 2-year PFS, similar to our Delta_rad_’s AUC of 0.74 in the training cohort [22]. Their study, however, lacked internal validation. Our findings, demonstrating robust prognostic performance in both training and validation cohorts, reinforce the promising role of T2WI radiomic signatures as effective tools for early prognostication and prediction of therapeutic outcomes in LACC. Such tools are essential not only for developing more targeted and effective treatment strategies but also for designing optimal patient follow-up plans, tailored to individual needs and prognoses.

Previous studies reporting temporal changes in specific radiomic features (from T2WI and/or apparent diffusion coefficient (ADC) maps) in LACC during CCRT, found that change in radiomic features during treatment yields better prediction of outcome than pretreatment radiomic features [12, 13, 14, 15]. This contrasts our finding that baseline tumor T2WI radiomics (reflected in Pre-CCRT_rad_) yield similar prognostic information to that from Delta_rad_. Of note, tumor segmentation, an integral part of radiomics, is often performed manually, leading to potential interobserver variability for the radiomic feature extraction [34]. Whereas interobserver agreement for primary tumor segmentations in the present study was excellent (Dice score of 0.85 and high intraclass correlation coefficients (ICC) for most features), this was much lower at mid-CCRT MRI (Dice score of 0.57 with lower ICCs). This mirrors trends observed in other studies regarding LACC tumor delineations and radiomic feature repeatability at pre- and post-4 weeks of CCRT MRI. Specifically, these studies reported a reduction in Dice scores (0.76 vs. 0.85 and 0.66 vs. 0.87 in post- vs. pre-CCRT MRIs, respectively) and a decrease in radiomic feature ICCs at post-CCRT MRIs [12, 13]. The lower agreement on tumor segmentations after CCRT can be attributed to several factors. Most of the patients (26/28 manually segmented by both radiologists) had a cervical sleeve at mid-CCRT MRI. This, combined with irradiative effects, complicates mid-CCRT assessment of LACC by altering normal anatomy, obscuring tissue planes, and diminishing the visibility of tumor boundaries. Furthermore, the smaller tumors, particularly seen in responders, could also explain some of the segmentation challenges [35]. Importantly, this could lead to less precise and reproducible radiomic feature extraction, potentially contributing to the apparently limited benefit of Delta_rad_ in the present study. This observation underscores the imperative need to address interobserver variability in radiomic studies in order to enhance the robustness, reproducibility, and universal applicability of results.

Our analysis identified distinct temporal changes in Delta_rad_- and Pre-CCRT_rad_ radiomic features between progressors and non-progressors, suggesting different treatment responses at a mesoscopic level within the tumors. We describe four key radiomic features (included in Delta_rad_ only [*n* = 1], Pre-CCRT_rad_ only [*n* = 2] or both [*n* = 1]) undergoing significant changes during CCRT, with markedly different patterns in patients with and without progression. All these features belong to the Gray Level Size Zone Matrix (GLSZM) category, which describes connected voxels sharing the same gray level intensities, reflecting textural tumor characteristics [10, 36].

To reduce the influence of large tumor size, a well-known adverse prognostic factor in LACC [26], our radiomic model deliberately excluded shape features. This strategy allowed us to focus on intrinsic tumor characteristics beyond dimensional assessments. Importantly, among the four key GLSZM features at baseline, one showed no correlation with pretreatment tumor diameter, whereas the remaining three were only weakly correlated, indicating their relative independence from size. Furthermore, despite progressors having larger tumor sizes at both pre- and mid-CCRT MRI, the relative size reduction was similar between progressors and non-progressors. This underscores that the identified delta radiomic patterns, capturing temporal mesoscale characteristics, are not simply surrogate markers of tumor shrinkage.

The association between tumor tissue heterogeneity, often considered to indicate an aggressive cancer phenotype [37], and quantitative radiomic imaging markers also reflecting tumor heterogeneity, is still not well understood [9]. Therefore, fully understanding the biological implications of the observed radiomic patterns poses a substantial challenge [9]. Notably, the more pronounced radiomic feature changes observed in progressors than in non-progressors suggest that these features may be specifically linked to tumor biology and treatment resistance in CC. Further research is needed that combines molecular, histopathological, genomic, and radiomic tumor data in order to unravel the underlying biological mechanisms that may be captured by delta radiomic profiling.

Limitations.

First, the relatively small study cohort and the lack of independent validation limit the robustness and generalizability of our predictive models. Future research should aim for larger LACC cohorts and include external validation to enhance clinical applicability. This approach would provide a more reliable estimate of the performance and potential clinical utility of radiomic signatures in practice. Second, our radiomic analysis was restricted to T2WI. Incorporating additional MRI sequences, particularly DWI, could potentially enhance the prognostic performance of our models. However, limited DWI data in our cohort (available for only 53/110 patients at both time points) precluded a more comprehensive image analysis. Third, the moderate Dice score for mid-CCRT segmentations, potentially reducing the robustness of the delta radiomic analyses, necessitated the adoption of stringent criteria for radiomic feature selection. While this rigorous approach may have slightly limited the predictive capacity of our models, it likely improved the reliability and transferability of our findings by ensuring that the chosen radiomic features were less affected by measurement artifacts or analysis inconsistencies. Lastly, our study’s multicenter setting inherently involved variability due to different MRI scanners and settings. Despite comprehensive normalization efforts, further research is essential to fully understand and mitigate the effects of this variability on radiomic feature analysis. This is a critical step to ensure the reliability and broad applicability of radiomic studies across various clinical environments.

## Conclusions

This study demonstrates that T2WI-based delta and pretreatment radiomic signatures hold significant promise for early prognostication in patients with LACC undergoing CCRT. Remarkably, these radiomic signatures outperform traditional prognostic markers, such as 2018 FIGO stage and baseline MRI assessments of maximum tumor diameter, for predicting PFS. To fully ascertain the clinical utility of these signatures, further validation using larger and independent patient cohorts is imperative. Importantly, future research should also delve into the biological mechanisms underlying radiomic signatures, a critical step for their clinical adoption and the integration of radiomic tumor profiling for clinical decision-making. This could pave the way for more tailored and effective treatment and follow-up strategies in LACC that improve patient outcomes.

## Electronic supplementary material

Below is the link to the electronic supplementary material.

Supplementary Material 1

## Data Availability

The datasets generated and analyzed during the current study are not publicly available due to privacy restrictions but are available from the corresponding author upon reasonable request.

## Abbreviations

AUC: Area under the curve
CC: Cervical cancerappears
CCRT: Concurrent chemoradiotherapy
CE T1WI: Contrast-enhanced T1-weighted imaging
CT: Computed tomography
DWI: Diffusion-weighted imaging
FDG-PET/CT: ^18^F-fluorodeoxyglucose positron emission tomography with computed tomography
FIGO: International Federation of Gynecology and Obstetrics
GLCM: Gray Level Co-occurrence Matrix
GLDM: Gray Level Dependence Matrix
GLRLM: Gray Level Run Length Matrix
GLSZM: Gray Level Size Zone Matrix
ICC: Intraclass correlation coefficient
IQR: Interquartile range
LACC: Locally advanced cervical cancer
MRI: Magnetic resonance imaging
NGTDM: Neighboring Gray Tone Difference Matrix
PFS: Progression-free survival
tdROC: Time-dependent receiver operating characteristics
T1WI: T1-weighted imaging
T2WI: T2-weighted imaging
