# Supplementary material for: MRI delta radiomics during chemoradiotherapy for prognostication in locally advanced cervical cancer
Source: BMC Cancer. 2025 Jan 22;25:122. doi: 10.1186/s12885-025-13509-1 (PMC11753090; doi:10.1186/s12885-025-13509-1)
Supplement: Supplementary file 1 — Supplementary Material 1 [file 12885_2025_13509_MOESM1_ESM.docx]

| **Supplementary Table 1** Clinical- and pathological characteristics for the locally advanced cervical cancer (LACC) patients included in this study (*n*_1_=110) and the entire cervical cancer cohort (*n*_2_=206) treated with concurrent chemoradiotherapy (CCRT) during the same period (2007–2022) at our hospital. | | | |
| --- | --- | --- | --- |
|  | Total study  cohort  (*n*_1_=110) | Entire CCRT cohort  (*n*_2_=206) | *p* |
| Age, years, median (IQR) [*n*_1_=110/*n*_2_=206] | 48 (39–57) | 50 (40–60) | 0.11 |
| BMI, kg/m^2^, median (IQR) [*n*_1_=110/*n*_2_=206] | 25 (22–28) | 25 (22–29) | 0.73 |
| Menopausal status, *n* (%) [*n*_1_=110/*n*_2_=205] |  |  | 0.08 |
| Pre- /perimenopausal | 67 (61) | 103 (50) |  |
| Postmenopausal | 43 (39) | 102 (50) |  |
| Maximum tumor diameter at pre-CCRT MRI^a^, *n* (%) [*n*_1_=110 /*n*_2_=172] |  |  | 0.54 |
| ≤2 cm | 2 (2) | 8 (4) |  |
| >2 and ≤4 cm | 23 (21) | 36 (18) |  |
| >4 cm | 85 (77) | 128 (62) |  |
| FIGO stage, *n* (%) [*n*_1_=110/*n*_2_=206] |  |  | 0.41 |
| I | 8 (7) | 22 (11) |  |
| II | 38 (35) | 69 (34) |  |
| III | 57 (52) | 93 (45) |  |
| IV | 7 (6) | 22 (11) |  |
| Histologic type, *n* (%) [*n*_1_=110/*n*_2_=206] |  |  | 0.73 |
| Squamous cell carcinoma | 91 (83) | 177 (86) |  |
| Adenocarcinoma | 17 (16) | 25 (12) |  |
| Other^b^ | 2 (2) | 4 (2) |  |
| Histologic grade, *n* (%) [*n*_1_=67/*n*_2_=130] |  |  | 0.82 |
| 1&2 | 51 (46) | 94 (46) |  |
| 3 | 16 (15) | 36 (18) |  |
| Progression, *n* (%) [*n*_1_=110/*n*_2_=206] |  |  | 0.24 |
| Yes | 35 (32) | 52 (25) |  |
| No | 75 (68) | 154 (75) |  |
| Dead from cervical cancer, *n* (%) [*n*_1_=110/*n*_2_=206] |  |  | 0.68 |
| Yes | 29 (26) | 49 (24) |  |
| No | 81 (74) | 157 (76) |  |
| *P* values refer to the Mann–Whitney U test for continuous variables and Fischer’s exact test for categorical variables. Significant *p* values are given in **bold**.  ^a^MRI-derived maximum tumor diameter, measured regardless of plane, later grouped into three categories.  ^b^Undifferentiated carcinoma or inconclusive biopsy.  BMI, body mass index; FIGO, International Federation of Gynecology and Obstetrics; IQR, interquartile range. | | | |

| **Supplementary Table 2** T2WI TSE acquisition parameters for the pelvic MRI examinations performed prior to treatment (pre-CCRT MRIs) (*n* = 110) and during concurrent chemoradiotherapy (mid-CCRT MRIs) (*n* = 110). Values are given as median (range). | | | | | | |
| --- | --- | --- | --- | --- | --- | --- |
|  |  | Siemens Healthineers 1.5T | GE Healthcare 1.5T | Philips Healthcare 1.5T | Siemens Healthineers 3.0T | Philips Healthcare 3.0T |
| pre-CCRT  MRI |  | *n*=33 | *n*=6 | *n*=21 | *n*=38 | *n*=12 |
| Ax/AxObl |  |  |  |  |  |  |
|  | TR^a^ [ms] | 3890 (2900–5870) | 3212 (2412–4365) | 4750 (2482-5415) | 4385 (4000–6940) | 4095 (3874–4984) |
|  | TE^a^ [ms] | 100 (84–114) | 84 (80–96) | 100 (90–130) | 94 (92–104) | 110 (100–110) |
|  | FA^a^ [degree] | 150 (125–180) | 160 (160–160) | 90 (90–90) | 150 (130–160) | 90 (90–90) |
|  | FOV (x, y) [mm^2^] | 200x200 (180x180–250x250) | 180x180 (180x180–200x200) | 200x200 (180x180–215x215) | 200x200 (173x173–200x200) | 180x180 (180x180–218x218) |
|  | Matrix shape (x, y) | 512x512 (256x256–640x640) | 512x512 (512x512–512x512) | 512x512 (256x256–528x528) | 384x384 (320x320–768x768) | 512x512 (480x480–512x512) |
|  | Slice thickness^a^ [mm] | 4.0 (3.0–5.0) | 3.0 (3.0–4.0) | 3.0 (3.0–5.0) | 3.0 (2.5–4.0) | 2.5 (2.5–4.0) |
|  | Interslice gap [mm] | 0.5 (0–0.6) | 0 (0–0.4) | 0.3 (0–1.0) | 0.3 (0–0.9) | 0.3 (0.3–0.4) |
|  | Number of slices | 30 (19–34) | 30 (25–40) | 26 (20–36) | 24 (22–40) | 35 (20–45) |
|  | Pixel size (x, y) [mm^2^] | 0.4x0.4 (0.3x0.3–0.8x0.8) | 0.4x0.4 (0.4x0.4–0.4x0.4) | 0.4x0.4 (0.3x0.3–0.8x0.8) | 0.5x0.5 (0.2x0.2–0.6x0.6) | 0.4x0.4 (0.4x0.4–0.5x0.5) |
|  | NEX^a^ | 2 (1–4) | 2 (2–3) | 6 (3–10) | 3 (2–3) | 2 (1–2) |
| mid-CCRT  MRI |  | *n*=63 | *n*=16 |  | *n*=31 |  |
| Ax/AxObl |  |  |  |  |  |  |
|  | TR^a^ [ms] | 4790 (3000–7010) | 4985 (2300–6385) |  | 4610 (3310–5450) |  |
|  | TE^a^ [ms] | 100 (71–121) | 77 (76–82) |  | 94 (91–94) |  |
|  | FA^a^ [degree] | 150 (130–180) | 110 (110–150) |  | 148 (128–160) |  |
|  | FOV (x, y) [mm^2^] | 200x200 (160x160–250x250) | 200x200 (200x200–200x200) |  | 200x200 (200x200–200x200) |  |
|  | Matrix shape (x, y) | 320x320 (256x256–512x512) | 512x512 (512x512–512x512) |  | 384x384 (320x320–384x384) |  |
|  | Slice thickness^a^ [mm] | 3.0 (3.0–5.0) | 3.0 (3.0–3.0) |  | 3.0 (3.0–4.0) |  |
|  | Interslice gap [mm] | 0.3 (0–1.0) | 0.3 (0.3–0.3) |  | 0.3 (0–0.8) |  |
|  | Number of slices | 27 (21–45) | 27 (27–30) |  | 25 (24–40) |  |
|  | Pixel size (x, y) [mm^2^] | 0.6 (0.3–0.8) | 0.4x0.4 (0.4x0.4–0.4x0.4) |  | 0.5x0.5 (0.5x0.5–0.6x0.6) |  |
|  | NEX^a^ | 2 (1–3) | 3 (2–3) |  | 3 (2–3) |  |
| ^a^Primary variables used in the linear regression model for radiomic feature normalization.  Secondary variables used in the linear regression model for feature normalization are derived from the primary variables listed in the table: anisotropy = slice thickness/pixel size (x); voxel volume = slice thickness × pixel size (x) × pixel size (y); FOV = Matrix shape (x × y) × pixel size (x × y).  Ax, axial; AxObl, axial oblique; CCRT, concurrent chemoradiotherapy; FA, flip angle; FOV, field of view; NEX, number of excitations; TE, time to echo; TR, repetition time; TSE, turbo spin echo; T2WI; T2-weighted imaging. | | | | | | |

| **Supplementary Table 3** List of extracted radiomic features from T2-weighted imaging (T2WI). The reproducibility of the radiomic features extracted from the segmentations performed by two radiologists in 28 randomly chosen patients was assessed by intraclass correlation coefficients (ICCs). Only radiomic features with an ICC >0.75 and a lower confidence interval (CI) boundary of ≥0.60 in both pre-CCRT and mid-CCRT MRIs were retained for further analysis (26/82; 32%) (given in **bold**). | | | | |
| --- | --- | --- | --- | --- |
|  | Pre-CCRT  MRIs  ICC | 95% CI | Mid-CCRT MRIs  ICC | 95% CI |
| First-order Energy | 0.85 | 0.71–0.93 | 0.79 | 0.40–0.92 |
| First-order Entropy | 0.94 | 0.88–0.97 | 0.63 | 0.34–0.81 |
| First-order Kurtosis | 0.92 | 0.84–0.96 | 0.39 | 0.03–0.66 |
| First-order Skewness | 0.92 | 0.84–0.96 | 0.58 | 0.28–0.78 |
| First-order Total Energy | 0.75 | 0.53–0.87 | 0.76 | 0.27–0.91 |
| First-order Uniformity | 0.94 | 0.87–0.97 | 0.47 | 0.14–0.71 |
| First-order Variance | 0.99 | 0.97–0.99 | 0.79 | 0.59–0.90 |
| **GLCM Autocorrelation** | **0.94** | **0.88–0.97** | **0.82** | **0.64–0.91** |
| GLCM Cluster Prominence | 1.00 | 1.00–1.00 | 0.35 | 0.00–0.64 |
| GLCM Cluster Shade | 1.00 | 0.99–1.00 | 0.55 | 0.23–0.76 |
| GLCM Cluster Tendency | 0.99 | 0.98–1.00 | 0.70 | 0.46–0.85 |
| GLCM Contrast | 0.89 | 0.78–0.95 | 0.62 | 0.33–0.81 |
| GLCM Correlation | 0.93 | 0.86–0.97 | 0.64 | 0.30–0.82 |
| GLCM Difference Average | 0.95 | 0.89–0.97 | 0.75 | 0.53–0.88 |
| GLCM Difference Entropy | 0.95 | 0.89–0.98 | 0.75 | 0.53–0.87 |
| GLCM Difference Variance | 0.86 | 0.72–0.93 | 0.61 | 0.31–0.80 |
| **GLCM Id** | **0.97** | **0.94–0.99** | **0.82** | **0.64–0.91** |
| **GLCM Idm** | **0.97** | **0.94–0.99** | **0.82** | **0.64–0.91** |
| GLCM Idmn | 0.65 | 0.38–0.82 | 0.60 | 0.30–0.79 |
| GLCM Idn | 0.77 | 0.56–0.88 | 0.71 | 0.44–0.86 |
| GLCM Imc1 | 0.98 | 0.95–0.99 | 0.77 | 0.55–0.89 |
| GLCM Imc2 | 0.91 | 0.81–0.96 | 0.42 | 0.08–0.68 |
| **GLCM Inverse Variance** | **0.95** | **0.89–0.97** | **0.81** | **0.64–0.91** |
| GLCM Joint Average | 0.94 | 0.88–0.97 | 0.78 | 0.58–0.89 |
| GLCM Joint Energy | 0.95 | 0.89–0.97 | 0.40 | 0.06–0.66 |
| GLCM Joint Entropy | 0.94 | 0.87–0.97 | 0.64 | 0.35–0.81 |
| GLCM MCC | 0.93 | 0.86–0.97 | 0.49 | 0.17–0.72 |
| GLCM Maximum Probability | 0.96 | 0.91–0.98 | 0.58 | 0.28–0.78 |
| GLCM Sum Average | 0.94 | 0.88–0.97 | 0.78 | 0.58–0.89 |
| GLCM Sum Entropy | 0.93 | 0.86–0.97 | 0.53 | 0.20–0.75 |
| GLCM Sum Squares | 0.99 | 0.98–0.99 | 0.68 | 0.42–0.84 |
| GLDM Dependence Entropy | 0.94 | 0.87–0.97 | 0.50 | 0.11–0.75 |
| GLDM Dependence Non-Uniformity | 0.95 | 0.89–0.98 | 0.74 | 0.26–0.89 |
| **GLDM Dependence Non-Uniformity Normalized** | **0.98** | **0.94–0.99** | **0.89** | **0.78–0.95** |
| **GLDM Dependence Variance** | **0.99** | **0.98–1.00** | **0.87** | **0.74–0.94** |
| GLDM Gray Level Non-Uniformity | 0.99 | 0.98–1.00 | 0.76 | 0.47–0.89 |
| **GLDM Gray Level Variance** | **0.99** | **0.97–0.99** | **0.79** | **0.60–0.90** |
| **GLDM High Gray Level Emphasis** | **0.95** | **0.89–0.98** | **0.84** | **0.68–0.92** |
| **GLDM Large Dependence Emphasis** | **0.98** | **0.94–0.99** | **0.87** | **0.73–0.94** |
| **GLDM Large Dependence High Gray Level Emphasis** | **0.96** | **0.92–0.98** | **0.91** | **0.82–0.96** |
| GLDM Large Dependence Low Gray Level Emphasis | 0.95 | 0.89–0.98 | 0.47 | 0.12–0.71 |
| GLDM Low Gray Level Emphasis | 0.94 | 0.88–0.97 | 0.55 | 0.23–0.76 |
| **GLDM Small Dependence Emphasis** | **0.94** | **0.84–0.98** | **0.94** | **0.88–0.97** |
| **GLDM Small Dependence High Gray Level Emphasis** | **0.92** | **0.84–0.96** | **0.91** | **0.81–0.96** |
| GLDM Small Dependence Low Gray Level Emphasis | 0.94 | 0.88–0.97 | 0.79 | 0.59–0.90 |
| GLRLM Gray Level Non-Uniformity | 0.99 | 0.98–0.99 | 0.75 | 0.43–0.89 |
| GLRLM Gray Level Non-Uniformity Normalized | 0.94 | 0.87–0.97 | 0.47 | 0.14–0.71 |
| GLRLM Gray Level Variance | 0.99 | 0.97–0.99 | 0.79 | 0.59–0.90 |
| **GLRLM High Gray Level Run Emphasis** | **0.95** | **0.89–0.98** | **0.84** | **0.68–0.92** |
| **GLRLM Long Run Emphasis** | **0.99** | **0.98–1.00** | **0.90** | **0.80–0.95** |
| **GLRLM Long Run High Gray Level Emphasis** | **0.97** | **0.93–0.98** | **0.82** | **0.66–0.92** |
| GLRLM Long Run Low Gray Level Emphasis | 0.96 | 0.92–0.98 | 0.52 | 0.19–0.75 |
| GLRLM Low Gray Level Run Emphasis | 0.94 | 0.88–0.97 | 0.54 | 0.22–0.76 |
| GLRLM Run Entropy | 0.94 | 0.87–0.97 | 0.49 | 0.14–0.73 |
| GLRLM Run Length Non-Uniformity | 0.95 | 0.89–0.98 | 0.75 | 0.29–0.90 |
| **GLRLM Run Length Non-Uniformity Normalized** | **0.97** | **0.88–0.99** | **0.87** | **0.74–0.94** |
| **GLRLM Run Percentage** | **0.98** | **0.91–0.99** | **0.88** | **0.76–0.94** |
| **GLRLM Run Variance** | **0.99** | **0.98–1.00** | **0.91** | **0.81–0.96** |
| **GLRLM Short Run Emphasis** | **0.98** | **0.92–0.99** | **0.87** | **0.75–0.94** |
| **GLRLM Short Run High Gray Level Emphasis** | **0.95** | **0.89–0.98** | **0.85** | **0.70–0.93** |
| GLRLM Short Run Low Gray Level Emphasis | 0.94 | 0.88–0.97 | 0.56 | 0.25–0.77 |
| GLSZM Gray Level Non-Uniformity | 0.97 | 0.93–0.98 | 0.73 | 0.24–0.89 |
| GLSZM Gray Level Non-Uniformity Normalized | 0.91 | 0.82–0.96 | 0.37 | 0.03–0.64 |
| **GLSZM Gray Level Variance** | **0.98** | **0.95–0.99** | **0.82** | **0.64–0.91** |
| **GLSZM High Gray Level Zone Emphasis** | **0.95** | **0.89–0.98** | **0.83** | **0.67–0.92** |
| GLSZM Large Area Emphasis | 0.99 | 0.98–1.00 | 0.61 | 0.32–0.80 |
| GLSZM Large Area High Gray Level Emphasis | 0.98 | 0.97–0.99 | 0.58 | 0.28–0.78 |
| GLSZM Large Area Low Gray Level Emphasis | 0.99 | 0.98–1.00 | 0.69 | 0.44–0.84 |
| GLSZM Low Gray Level Zone Emphasis | 0.94 | 0.88–0.97 | 0.46 | 0.12–0.71 |
| GLSZM Size Zone Non-Uniformity | 0.94 | 0.88–0.97 | 0.52 | 0.14–0.76 |
| **GLSZM Size Zone Non-Uniformity Normalized** | **0.98** | **0.97–0.99** | **0.93** | **0.85–0.97** |
| **GLSZM Small Area Emphasis** | **0.98** | **0.95–0.99** | **0.92** | **0.84–0.96** |
| **GLSZM Small Area High Gray Level Emphasis** | **0.95** | **0.90–0.98** | **0.88** | **0.75–0.94** |
| GLSZM Small Area Low Gray Level Emphasis | 0.90 | 0.80–0.95 | 0.50 | 0.17–0.73 |
| GLSZM Zone Entropy | 0.98 | 0.95–0.99 | 0.70 | 0.24–0.87 |
| **GLSZM Zone Percentage** | **0.92** | **0.78–0.97** | **0.94** | **0.87–0.97** |
| GLSZM Zone Variance | 0.99 | 0.98–1.00 | 0.61 | 0.32–0.80 |
| NGTDM Busyness | 0.96 | 0.91–0.98 | 0.80 | 0.56–0.91 |
| NGTDM Coarseness | 0.85 | 0.70–0.93 | 0.71 | 0.27–0.88 |
| NGTDM Complexity | 0.92 | 0.83–0.96 | 0.35 | 0.00–0.64 |
| NGTDM Contrast | 0.96 | 0.92–0.98 | 0.51 | 0.18–0.74 |
| NGTDM Strength | 0.70 | 0.45–0.85 | 0.39 | 0.02–0.66 |
| CI, confidence interval; CCRT; concurrent chemoradiotherapy; GLCM, Gray Level Co-occurrence Matrix; GLDM, Gray Level Dependence Matrix; GLRLM, Gray Level Run Length Matrix; GLSZM, Gray Level Size Zone Matrix; NGTDM, Neighboring Gray Tone Difference Matrix. | | | | |

| **Supplementary Table 4** Correlation coefficients (*r*_S_)* between MRI-derived maximum tumor diameter measured at pre- and mid-CCRT time points and the corresponding radiomic features included in the signatures Delta_rad_ and Pre-CCRT_rad_. | | |
| --- | --- | --- |
|  | | Pre-CCRT MRI-derived  tumor diameter,  *r*_S_ |
| Pre-CCRT values of radiomic features within Delta_rad_ and Pre-CCRT_rad_ | |  |
| GLCM Autocorrelation^a^ | (f1) | **0.25*** |
| GLCM Inverse Variance^a^ | (f2) | **0.20*** |
| GLDM Dependence Non-Uniformity Normalized^b^ | (f3) | **-0.48**** |
| GLDM Dependence Variance^a, b^ | (f4) | **0.41**** |
| GLDM Gray Level Variance^a, b^ | (f5) | 0.03 |
| GLRLM Long Run Emphasis^b^ | (f6) | 0.14 |
| GLSZM Size Zone Non-Uniformity Normalized^a, b^ | (f7) | 0.18 |
| GLSZM Small Area Emphasis^b^ | (f8) | **0.20*** |
| GLSZM Small Area High Gray Level Emphasis^b^ | (f9) | **0.23*** |
| GLSZM Zone Percentage^a^ | (f10) | **-0.36**** |
|  | | Mid-CCRT MRI-derived  tumor diameter,  *r*_S_ |
| Mid-CCRT values of radiomic features within Delta_rad_ | |  |
| GLCM Autocorrelation | (f1) | 0.0002 |
| GLCM Inverse Variance | (f2) | **0.37**** |
| GLDM Dependence Variance | (f4) | **0.51**** |
| GLDM Gray Level Variance | (f5) | **-0.26*** |
| GLSZM Size Zone Non-Uniformity Normalized | (f7) | -0.002 |
| GLSZM Zone Percentage | (f10) | **-0.54**** |
| *Spearman rank correlation, *p*<0.05* and *p*<0.001** given in **bold.**  ^a^Features within Delta_rad_  ^b^Features within Pre-CCRT_rad_  CCRT, concurrent chemoradiotherapy; GLCM, Gray Level Co-occurrence Matrix; GLDM, Gray Level Dependence Matrix; GLSZM, Gray Level Size Zone Matrix. | | |

| **Supplementary Table 5** AUC with 95% CI for predicting 5-year progression-free survival (PFS) in locally advanced cervical cancer (LACC) patients treated with CCRT. | | | | |
| --- | --- | --- | --- | --- |
|  | Training cohort  (*n*=73) | | Validation cohort  (*n*=37) | |
|  | AUC_T_ | (95% CI) | AUC_V_ | (95% CI) |
| Delta_rad_ | 0.74 | (0.61–0.87) | 0.79 | (0.59–0.99) |
| Pre-CCRT_rad_ | 0.72 | (0.59–0.86) | 0.75 | (0.52–0.98) |
| FIGO^a^ | 0.61 | (0.48–0.74) | 0.61 | (0.39–0.83) |
| Tumor_max_^b^ | 0.58 | (0.49–0.67) | 0.65 | (0.51–0.79) |
| MRI-measured maximum tumor diameter reduction, % | 0.50 | (0.33–0.66) | 0.49 | (0.23–0.76) |
| Manually segmented tumor volume at pre-CCRT MRI | 0.58 | (0.43–0.73) | 0.62 | (0.37–0.88) |
| Tumor volume reduction, % | 0.50 | (0.34–0.67) | 0.51 | (0.23–0.78) |
| ^a^ FIGO stage (I–IV)  ^b^Maximum tumor diameter at pre-CCRT MRI (≤2; >2 and ≤4; >4 cm).  AUC, Area under the time-dependent receiver operating characteristic (tdROC) curves; CI, confidence interval; CCRT, concurrent chemoradiotherapy; FIGO, International Federation of Gynecology and Obstetrics. | | | | |

| **Supplementary Table 6** Association between radiomic features (extracted at pre-CCRT and mid-CCRT MRI, and the temporal change (Δfeature)) and progression in locally advanced cervical cancer (LACC) patients treated with CCRT. | | | | | |
| --- | --- | --- | --- | --- | --- |
| Radiomic features | Non-progressors  *(n*=75) | | Progressors  *(n*=35) | |  |
|  | Median | (IQR) | Median | (IQR) | *p* |
| **Pre-CCRT** |  |  |  |  |  |
| GLSZM Size Zone Non-Uniformity Normalized | -0.104 | (1.407) | 0.376 | (1.184) | **0.03** |
| GLSZM Zone Percentage | -0.175 | (0.752) | -0.286 | (0.907) | 0.59 |
| GLSZM Small Area Emphasis | -0.067 | (1.334) | 0.347 | (0.850) | **0.03** |
| GLSZM Small Area High Gray Level Emphasis | -0.122 | (1.410) | 0.264 | (1.172) | **0.03** |
|  |  |  |  |  |  |
| **Mid-CCRT** |  |  |  |  |  |
| GLSZM Size Zone Non-Uniformity Normalized | -0.099 | (1.260) | -0.503 | (0.963) | 0.10 |
| GLSZM Zone Percentage | 0.095 | (1.067) | -0.375 | (1.049) | **0.006** |
| GLSZM Small Area Emphasis | 0.005 | (1.086) | -0.318 | (0.780) | 0.19 |
| GLSZM Small Area High Gray Level Emphasis | -0.373 | (0.717) | -0.310 | (1.018) | 0.55 |
|  |  |  |  |  |  |
| **Δfeature** |  |  |  |  |  |
| GLSZM Size Zone Non-Uniformity Normalized | -0.242 | (1.534) | -0.883 | (1.741) | **0.01** |
| GLSZM Zone Percentage | 0.215 | (1.242) | -0.097 | (1.247) | **0.03** |
| GLSZM Small Area Emphasis | -0.172 | (1.604) | -0.861 | (1.444) | **0.02** |
| GLSZM Small Area High Gray Level Emphasis | -0.152 | (1.597) | -0.646 | (1.150) | **0.009** |
| *P* values refer to Mann Whitney U test. Significant *p* values are given in **bold.**  CCRT, concurrent chemoradiotherapy; GLSZM, Gray Level Size Zone Matrix; IQR, interquartile range (given as Q3-Q1). | | | | | |
